# Effect of the implantoplasty techniques on the fracture resistance of dental implants. Systematic review and meta-analysis

**DOI:** 10.3389/fdmed.2025.1568465

**Published:** 2025-05-15

**Authors:** Giovanni Pissu, Javier Flores-Fraile, Álvaro Zubizarreta-Macho, José María Montiel-Company, Ana Belén Lobo-Galindo

**Affiliations:** ^1^ Faculty of Dentistry, Alfonso X. El Sabio University, Madrid, Spain; ^2^ Department of Surgery, Faculty of Medicine and Dentistry, University of Salamanca, Salamanca, Spain; ^3^ Department of Stomatology, Faculty of Medicine and Dentistry, Universitat de Valéncia, Valencia, Spain

**Keywords:** implantoplasty periimplant disease, dental implants, fracture resistance, failure strength, implants

## Abstract

The aim of this systematic review and meta-analysis was to analyze and compare the effect of diamond drill, tungsten carbide drill and ultrasound tips for implantoplasty procedures on the fracture resistance of dental implants affected by periimplant disease.

**Materials and methods:**

This systematic review of the scientific literature and meta-analysis was carried out based on the recommendations of the Preferred Reporting Items for Systematic Reviews and Meta-Analyses (PRISMA), analyzing all studies that evaluated the fracture resistance of dental implants submitted to implantoplasty through diamond drill, tungsten carbide drill and ultrasound tips for implantoplasty procedures, comparing with the fracture resistance values of non-treated dental implants. A total of 4 databases were searched in the literature: Pubmed, Scopus, Cochrane and Web of Science. After eliminating duplicate articles and applying certain inclusion criteria, a total of 9 articles were selected and compared using the random effects model and inverse variance method. The significance of the effect size was measured with the *z* test, the heterogeneity using the *Q* test and the *I*^2^ and publication bias was analyzed using the trim-and-fill method

**Results:**

The difference in means between the treatment and control groups was estimated as the effect size, obtaining a statistically significant difference of −232.01 MPa., with a 95% confidence interval of the difference between −417.3 and −44.71 (*z* test = −2.43; *p*-value = 0.015). The meta-analysis has presented high heterogeneity with an *I*^2^ = 99.3% and a *Q* test = 2,195.7; *p*-value < 0.001. No significant differences were found between the three subgroups with the *Q* test = 0.20: *p*-value = 0.903.

**Conclusions:**

the tungsten carbide drills resulted in less fracture resistance loss than the diamond and tungsten carbide drills for the dental implants submitted to implantoplasty procedures.

**Systematic Review Registration**: [http://www.prisma-statement.org], identifier [INPLASY202460018].

## 1 Introduction

Peri-implantitis is a very common disease associated with dental implants (1). The origin of peri-implant disease has a multifactorial etiology, and the greatest risk factor is the accumulation of bacterial plaque (2). It presents two phases, mucositis and peri-implantitis, the first being an inflammation of the soft tissues around the implant and in the second there is an increase in the inflammatory response and a loss of bone around the implant with the formation of moderate or severe hose defects depending on the stage and evolution of peri-implantitis (2). If peri-implant disease is not treated this can lead to future loss of the implant (3).

There are different ways to treat peri-implantitis, we can divide them into surgical (resective and regenerative) and non-surgical therapies (4, 5).

Non-surgical therapies are the least effective in blocking the progression of moderate or severe peri-implantitis (6, 7). Surgical therapies seem to be the most predictable but depend a lot on the skill of the professional, the oral area where the implants are located and also their inclination (8, 9).

Implantoplasty is a resective surgical technique with which, by using burs or instruments, we eliminate the threads and roughness of the dental implant that have been exposed, this because the surface of the implant has been designed with a roughness that favors osseointegration with the bone. But it also favors the adhesion of the biofilm of the oral microbiota if exposed, increasing the inflammatory response of the tissues. With implantoplasty, it is possible to decontaminate the implant surface, eliminate exposed threads and reduce the roughness of the implant (going from a roughness of Ra < 0.4 *µ*m to one of Ra < 0.2 *µ*m) (10, 11).

The implantoplasty protocol is performed using turbine or tungsten or diamond burs using a sequence of burs from the coarsest grain to the finest grain, ending with Arkansas burs or silicone polishing burs (10–12).

Among the various disadvantages of implantoplasty we find the increased risk of fracture of the implants since it modifies both the structure, both the macro and the microstructure, as well as the thickness of the dental implant (13).

In this article we are going to evaluate whether implantoplasty treatment can increase the risk of dental implant fracture by analyzing the influence of the procedures during implantoplasty on the fracture resistance of dental implants.

The aim of this systematic review and meta-analysis was to analyze and compare the effect of diamond drill, tungsten carbide drill and ultrasound tips for implantoplasty procedures on the fracture resistance of dental implants affected by periimplant disease, with a null hypothesis (H_0_) stating that there are not statistically significant differences between the fracture resistance of dental implants affected by periimplant disease submitted to implantoplasty procedures through diamond drill, tungsten carbide drill and ultrasound tips.

## 2 Materials and methods

### 2.1 Study design

The literature review was conducted following guidelines for systematic reviews and meta-analyses in accordance with PRISMA (Preferred Reporting Items for Systemic Reviews and Meta-Analyses http://www.prisma-statement.org (accessed on 31 May 2024); International Platform of Registered Systematic Review and Meta-analysis Protocols (INPLASY) registration number: INPLASY202460018 (DOI number: 10.37766/inplasy2024.6.0018). The review also complied with the PRISMA 2020 Checklist and was performed in accordance with current recommendations regarding systematic reviews and meta-analyses. The population, intervention, comparison, and outcome (PICO) question was "The impact of the implantoplasty technique on the fracture resistance on the dental implant", with the following components: Population: dental implants subjected to implantoplasty technique; Intervention: implantoplasty; Comparison: implantoplasty technique with diamond drills, tungsten drills and AIR SCALER; and Outcome: fracture resistance.

An electronic search was carried out using the following databases: PubMed, Scopus, Cochrane, and Web of Sciences. The search assessed all the literature published internationally through to March 2024. Five medical subject heading (MeSH) terms were included in the search: “Implantoplasty”; “Periimplantitis”; “fracture resistance”; “failure strength”; “*in vitro*”. One Boolean operator was applied ("AND"). These search terms were applied as follows: [("implantoplasty") AND ("Periimplantitis") AND ("fracture resistance") AND ("failure strength") AND ("*in vitro*")]. Two different researchers (G.P.; A.Z.M.) searched the databases simultaneously. The inclusion and exclusion criteria were applied to titles, and a single researcher (G.P.) extracted the data regarding the relevant variables. A.Z.M. conducted the systematic review, and two researchers who had not participated in the selection process (A.Z.M.; G.P.) performed the subsequent meta-analysis.

The inclusion criteria were as follows: experimental trials (ETs). In all the implants they simulated a bone loss of half their length and were subjected to one of the implantoplasty techniques and then subjected or not to the loading cyclic test and to study the fracture resistance they used a universal servo hydraulic mechanical testing machine and the implant with a vertical angle of 30° Studies were not restricted by language or year of publication. The exclusion criteria were as follows: systematic literature reviews, prospective and retrospective randomized clinical trials, clinical cases, and editorials. The following data were recorded: author, year, title, journal, sample size (*n*). The results obtained from studies that analyzed the impact of the implatoplasty technique on the fracture resistance on the dental implants.

The Current Research Information System (CRIS) scale was used to assess the methodological quality of the selected *in vitro* studies, which is composed of four items that analyze the sample preparation, the randomization and blinding procedures and the statistical test. The best-rated studies were those that met all the concepts (14).

The meta-analysis will be carried out using the random effects model and inverse variance method. The significance of the effect size measured as a difference in means will be assessed with the *z* test. The heterogeneity of the meta-analysis will be analyzed using the *Q* test and the *I*^2^. For the difference between the subgroups, the *Q* test will be used, with the random effects model. The level of significance for all tests has been established at *p* < 0.05. The subgroup meta-analysis will be represented in a forest plot.

Publication bias will be analyzed using the funnel plot skew adjustment trim-and-fill method that adds new studies.

## 3 Results

### 3.1 Flow diagram

**Figure 1:**
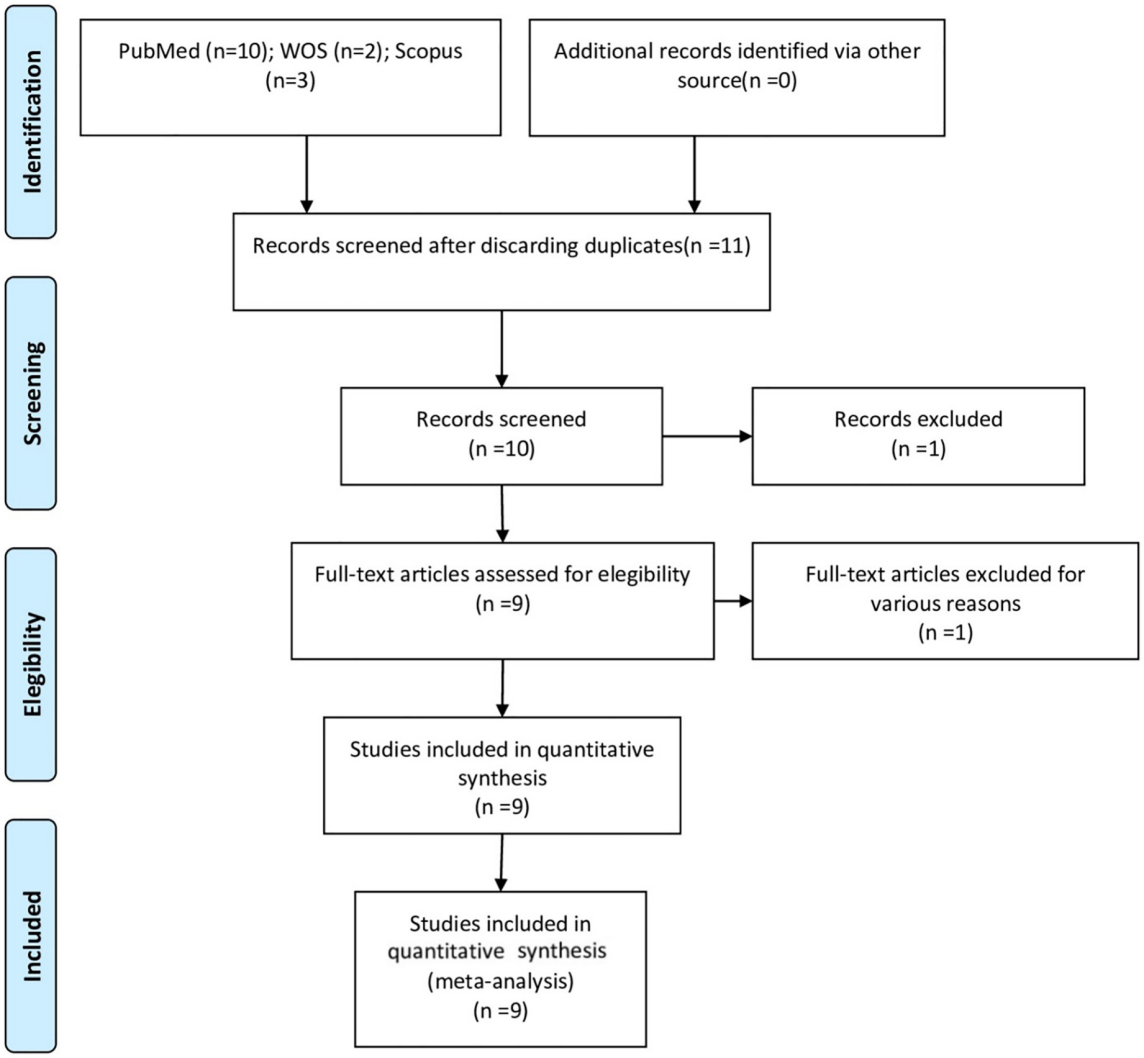
Preferred reporting items for systematic reviews and meta-analyses (PRISMA) flow diagram.

10 articles in PubMed, 2 articles in Web of Sciences and 3 articles in Scopus were found after the initial search. Of these 15 works, 2 duplicates were discarded. After assessing study titles and abstracts, another 1 were eliminated, after which 10 remained. An additional 1 were rejected for failing to fulfill the inclusion criteria by either not including the fracture strength or not meeting the minimum sample size. After this selection process, a total of 9 articles were selected for final qualitative synthesis. Nine articles were ultimately assessed in the quantitative analysis, as they met all the selection criteria (Figure 1).

### 3.2 Qualitative analysis

Of the 9 articles included, all were experimental trials. The studies showed a sample size ranged from as low as 15 in the study by Jorio et al. (2021) (15), to as high as 32 in Chan's study in 2013 (16) (Table 1).

**Table 1:** Qualitative analysis of articles forming part of the systematic review.

Author (Year)	Study type	Sample (*n*)	Variable	Fracture resistance
Bertl K et al. (16)	ET	20	Fracture resistance	870 ± 18 N
Chan H et al. (18)	ET	32	Fracture resistance	430,4 ± 26,8 N
Costa-Berenguer et al. (17)	ET	20	Fracture resistance	880 ± 193,7 N
Jorio et al. 2021 (15)	ET	15	Fracture resistance	1,645 ± 51 N
Leitão-Almeida B et al. (20)	ET	16	Fracture resistance	752,12 ± 186,13 N
Leitão-Almeida B et al. 2020 (19)	ET	16	Fracture resistance	621,68 ± 186,28 N
Shah SD et al. (21)	ET	17	Fracture resistance	665 ± 26 N
Sivolella D et al. (22)	ET	12	Fracture resistance	1,510 ± 170 N
Tsampli A et al. (23)	ET	20	Fracture resistance	739 ± 34 N

ET, controlled trial.

### 3.3 Quality assessment

Table 2 shows the results of the methodological quality assessment using the CRIS scale. All articles selected showed 2 point at the CRIS scale, resulting poor methodological quality. Quality scores were most often compromised by failure to fulfill criteria related to the randomization and blinding process.

**Table 2:** Methodological quality assessment as per the current research information system (CRIS) scale.

Author (Year)	Sample preparation and handling	Allocation sequence and randomization process	Whether the evaluators were blinded	Statistical analysis	Score
Costa-Berenguer et al. (17)	Yes	No	No	Yes	2
Bertl K et al. (16)	Yes	No	No	Yes	2
Chan H et al. (18)	Yes	No	No	Yes	2
Jorio et al. (15)	Yes	No	No	Yes	2
Leitão-Almeida B et al. (19)	Yes	No	No	Yes	2
Leitão-Almeida B et al. (20)	Yes	No	No	Yes	2
Shah SD et al. (21)	Yes	No	No	Yes	2
Sivolella D et al. (22)	Yes	No	No	Yes	2
Tsampli A et al. (23)	Yes	No	No	Yes	2

### 3.4 Quantitative analysis

10 studies with 16 comparisons have been combined using the random effects model and inverse variance method, to estimate the fracture resistance of implants subjected to static loading and implantoplasty. The total number of implants in the treatment groups was 132 and in the control group 120. The difference in means between the treatment and control groups was estimated as the effect size, obtaining a statistically significant difference of −232.01 MPa., with a 95% confidence interval of the difference between −417.3 and −44.71 (*z* test = −2.43; *p*-value = 0.015). The meta-analysis has presented high heterogeneity with an *I*^2^ = 99.3% and a *Q* test = 2,195.7; *p*-value < 0.001.

**Figure 2:**
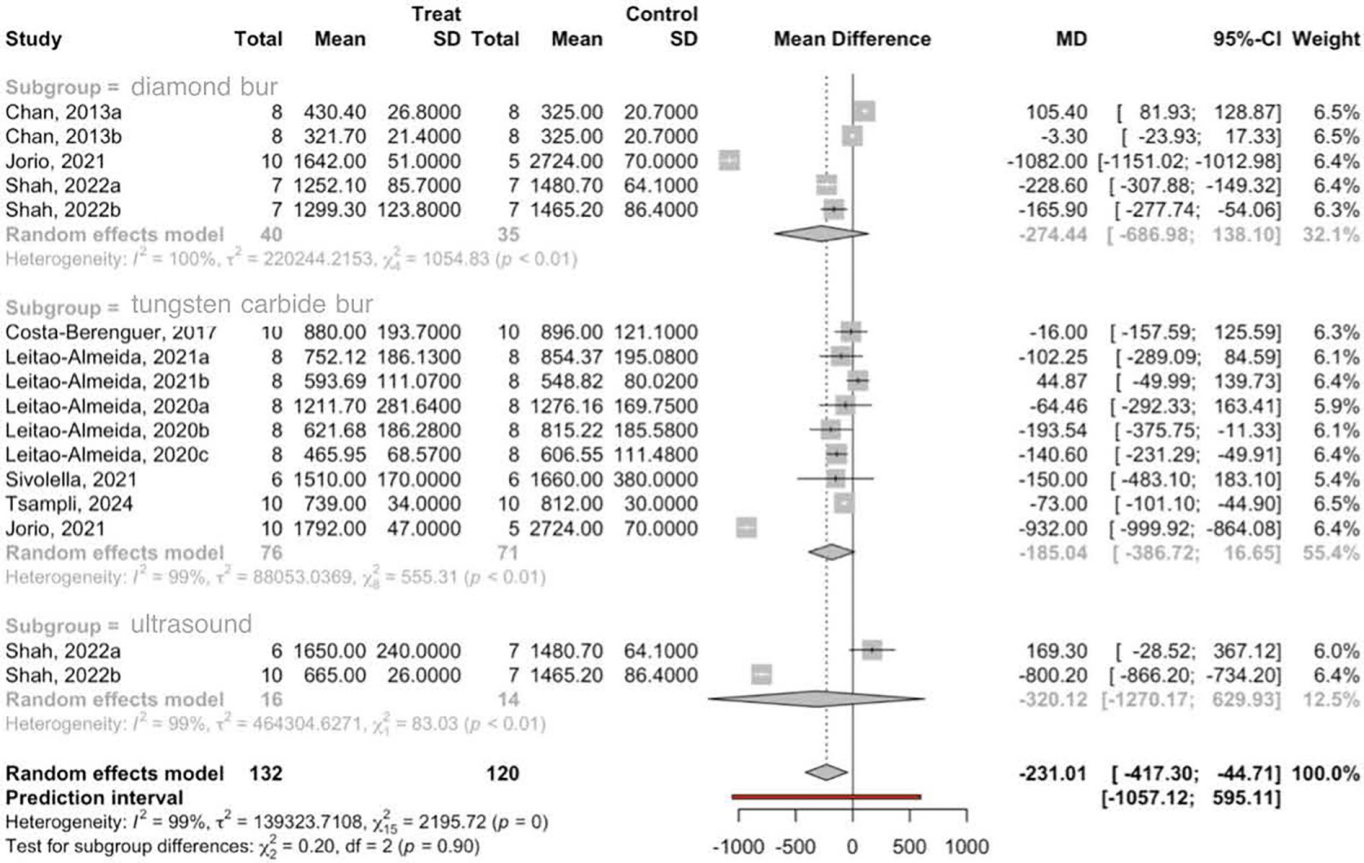
Forest plot of the subgroup meta-analysis of MPa fracture resistance.

A subgroup analysis was performed, differentiating type of drill and use of ultrasound, to assess the possible source of heterogeneity. In this way, it has been estimated that in the diamond bur group the fracture resistance has decreased −274.4 MPa, the tungsten bur group −185.04 MPa and the ultrasonic group −320.12 MPa. In all subgroups, fracture resistance has decreased, although not in a statistically significant way. No significant differences were found between the three subgroups with the *Q* test = 0.20: *p*-value = 0.903, which indicates that the heterogeneity is not due to the subgroup (Figure 2).

### 3.5 Publication bias

**Figure 3:**
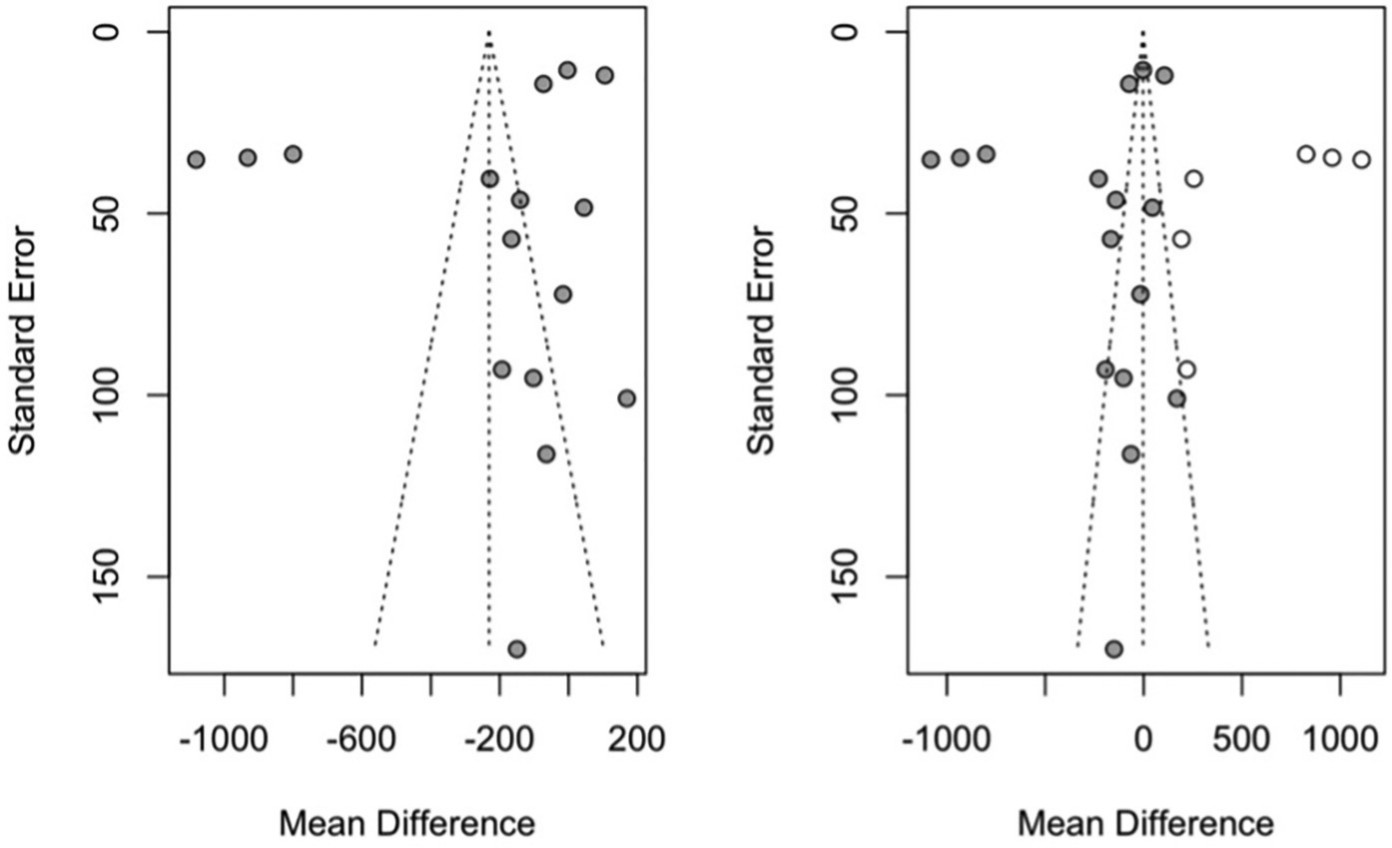
Funnel plots of the meta-analysis of fracture resistance using the trim-and-fill method with the initial estimate (left funnel plot) and the estimate with the studies added as white points (right funnel plot).

Publication bias has been studied using the Trim-and-fill method of adjusting the asymmetry of the funnel plot, adding 6 studies the effect size is estimated at −2.62 MPa, with a 95% CI > between—226.3 and 221.1 (*z* test = −0.02; *p*-value = 0.982). This shows that the meta-analysis is exposed to a possible publication bias that would nullify the estimated effect (Figure 3).

## 4 Discussion

The results obtained in the present study reject the null hypothesis (H_0_), which holds that there are not statistically significant differences between the fracture resistance of dental implants affected by periimplant disease submitted to implantoplasty procedures through diamond drill, tungsten carbide drill and ultrasound tips.

From the results we have obtained we see that implants subjected to implantoplasty have a lower resistance to fracture than implants without implantoplasty. In the present study we have also evaluated whether the resistance to fracture changes with different implatoplasty drills and we have seen that with tungsten drills the resistance is greater than the general average, while with diamond drills there is an increase in the risk of fracture and finally with ultrasonic drills are those that generate the highest risk of fracture, although we have little data on this type of implantoplasty technique. In the 2022 study by Jorrio et al, the results obtained show that there is a decrease in fracture resistance but not as great as what we have obtained in our study. This may be because in Jorrio's study they used all standard implants of 4.1 mm diameter while we have evaluated the resistance in different types of implants with different diameters and this may be the reason for the very discrepant data (14). The same also happens with the results obtained in the study carried out by Costa-Berenguer et al. in 2017 in which they evaluated the fracture resistance of standard 4.1 mm external connection implants in which an implantoplasty was performed with tungsten burs. confirming our hypothesis that depending on the size of the implant, implantoplasty generates a lower or higher risk of fracture (15). This difference in change in fracture resistance is described in the study by Bertl et al. in 2020 in which they evaluated how much implantoplasty can affect fracture resistance in Narrow (3.3 mm) and standard (4.1 mm) implants., the results show that Narrow implants subjected to implantoplasty have a very large decrease in fracture resistance compared to standard ones (17). Another study that describes this change in the risk of fracture is that of Chan H et al. from 2013 in which it was evaluated how much the fracture resistance changes in implants with implantoplasty between Narrow implants and 4.6 mm implants, in this case we see that. The fracture resistance of the implants is 4.6, although with implantoplasty it is twice the resistance of the Narrow implants (16). Another factor that must be taken into account when assessing the fracture resistance of implants, especially when an implantoplasty has been performed, is the vertical bone loss around the implant and this is demonstrated by Leitao-Almeida et al. in their two studies, one carried out in 2020 and the other in 2021 in which it has been evaluated how the fracture resistance of implants can change with implantoplasty (18, 19). In the 2020 study, the proportion of bone loss crown of the implant was evaluated with proportions 2:1, 2.5:1 and 3:1 in implants of the same diameter and same length and the results obtained confirmed that the greater the bone loss around the implant lower resistance to implant fracture18. In the study, always by the same author but from 2021, it was always evaluated whether vertical bone loss affects fracture resistance or not, but this time without the crowns, only with the healing abudment, subdividing the implants into two groups, some with bone loss of 3.5 mm and the other group with bone loss of 7 mm and the results obtained have shown that implants with greater bone loss have lower resistance to fracture (19). Among the various factors that must be considered are the material with which the implant is made, as there are new titanium alloys. Comparing the studies by Costa-berenguer et al. from 2017 that evaluated the fracture resistance of 4.1 × 10 mm grade IV titanium implants with bone loss of 5 mm and the study by Shah SD et al. in 2022 in which did the same study but evaluating the resistance of 4.1 × 10 mm implants but of the roxolid alloy from the straumann group, an alloy that has 85% titanium and 15% zirconium, with a bone loss of 5 mm, from the results obtained from the Two studies show that the Fmax is 200 N more with the roxolid alloy and thanks to these data they confirm that the roxolid alloy has a greater fracture resistance than titanium (15–20). Fracture resistance can vary depending on the milling system and also on the operator's experience, which is why in our study we have selected all data where the implantoplasty has been performed by machinery to try to make the data as homogeneous as possible, and thanks to the studies by Sivolella et al. 2021 and Tsampli et al. 2024 which evaluated how fracture resistance can vary depending on the implant milling system between tungsten, diamond and ultrasonic drills. From the results obtained, it can be seen that implantoplasty with tungsten drills generates a lower risk of fracture than compared to the other two systems, just like our results (21–23).

## 5 Conclusion

The conclusion derived from the present study is that the tungsten carbide drills resulted in less fracture resistance loss than the diamond and tungsten carbide drills for the dental implants submitted to implantoplasty procedures.

## Data Availability

The original contributions presented in the study are included in the article/Supplementary Material, further inquiries can be directed to the corresponding author.

## References

[1] Mir-Mari J Mir-Orfila P Figueiredo R Valmaseda-Castellón E Gay-Escoda C. Prevalence of peri-implant diseases. A cross-sectional study based on a private practice environment. J Clin Periodontol. (2012) 39:490–4. 10.1111/j.1600-051X.2012.01872.x 22486273

[2] Jepsen S Berglundh T Genco R Aass AM Demirel K Derks J Primary prevention of peri-implantitis: managing peri-implant mucositis. J Clin Periodontol. (2015) 42 Suppl 16:S152–7. 10.1111/jcpe.12369 25626479

[3] García-García M Mir-Mari J Benic GI Figueiredo R Valmaseda-Castellón E. Accuracy of periapical radiography in assessing bone level in implants affected by peri-implantitis: a cross-sectional study. J Clin Periodontol. (2016) 43:85–91. 10.1111/jcpe.12491 26660842

[4] Smeets R Henningsen A Jung O Heiland M Hammächer C Stein JM. Definition, etiology, prevention and treatment of peri-implantitis-a review. Head Face Med. (2014) 10:34. 10.1186/1746-160X-10-34 25185675 PMC4164121

[5] Schwarz F John G Schmucker A Sahm N Becker J. Combined surgical therapy of advanced peri-implantitis evaluating two methods of surface decontamination: a 7-year follow-up observation. J Clin Periodontol. (2017) 44:337–42. 10.1111/jcpe.12648 28101947

[6] Renvert S Roos-Jansaker AM Claffey N. Non-surgical treatment of peri-implant mucositis and peri-implantitis: a literature review. J Clin Periodontol. (2008) 35:305–15. 10.1111/j.1600-051X.2008.01276.x 18724858

[7] Estefanía-Fresco R García-de-la-Fuente AM Egaña-Fernández-Valderrama A Bravo M Aguirre-Zorzano LA. One-year results of a non-surgical treatment protocol for peri-implantitis. A retrospective case series. Clin Oral Implants Res. (2019) 30:702–12. 10.1111/clr.13456 31090974

[8] Karlsson K Derks J Håkansson J Wennström JL Petzold M Berglundh T. Interventions for peri-implantitis and their effects on further bone loss. A retrospective analysis of a registry-based cohort. J Clin Periodontol. (2019) 46:872–9. 10.1111/jcpe.13129 31077421

[9] Esposito M Grusovin MG Kakisis I Coulthard P Worthington HV. Interventions for replacing missing teeth: treatment of perimplantitis. Cochrane Database Syst Rev. (2008) (2):CD004970. 10.1002/14651858.CD004970.pub3 18425907

[10] Esteves Lima RP Abreu LG Belém FV Pereira GHM Brant RA Costa FO. Is implantoplasty efficacious at treating peri-implantitis? A systematic review and meta-analysis. J Oral Maxillofac Surg. (2021) 79:2270–9. 10.1016/j.joms.2021.06.015 34245700

[11] Albrektsson T Wennerberg A. Oral implant surfaces: Part 1 - review focusing on topographic and chemical properties of different surfaces and *in vivo* responses to them. Int J Prosthodont. (2004) 17(5):536–43. 15543910

[12] Lasserre JF Brecx MC Toma S. Implantoplasty versus glycine air abrasion for the surgical treatment of peri-implantitis: a randomized clinical trial. Int J Oral Maxillofac Implants. (2020) 35:197. 10.11607/jomi.6677 31923303

[13] Schwarz F John G Becker J. The influence of implantoplasty on the diameter, chemical surface composition, and biocompatibility of titanium implants. Clin Oral Investig. (2017) 21:2355–61. 10.1007/s00784-016-2030-x 28012063

[14] Biesenbender S Petersohn S Thiedig C. Using current research information systems (CRIS) to showcase national and institutional research (potential): research information systems in the context of open science. Procedia Comput Sci. (2019) 146:142–55. 10.1016/j.procs.2019.01.089

[15] Jorio IC Stawarczyk B Attin T Schmidlin PR Sahrmann P. Reduced fracture load of dental implants after implantoplasty with different instrumentation sequences. An *in vitro* study. Clin Oral Implants Res. (2021) 32(8):881–92. 10.1111/clr.13754 34031921

[16] Bertl K Isidor F von Steyern PV Stavropoulos A. Does implantoplasty affect the failure strength of narrow and regular diameter implants? A laboratory study. Clin Oral Investig. (2021) 25(4):2203–11. 10.1007/s00784-020-03534-8 32893312 PMC7966130

[17] Costa-Berenguer X García-García M Sánchez-Torres A Sanz-Alonso M Figueiredo R Valmaseda-Castellón E. Effect of implantoplasty on fracture resistance and surface roughness of standard diameter dental implants. Clin Oral Implants Res. (2018) 29(1):46–54. 10.1111/clr.13037 28736922

[18] Chan H Oh W Ong HS Fu JH Steigmann M Sierraalta M Impact of implantoplasty on strength of the implant-abutment complex. Int J Oral Maxillofac Implants. (2013) 28(6):1530–5. 10.11607/jomi.3227 24278921

[19] Leitão-Almeida B Camps-Font O Correia A Mir-Mari J Figueiredo R Valmaseda-Castellón E. Effect of crown to implant ratio and implantoplasty on the fracture resistance of narrow dental implants with marginal bone loss: an *in vitro* study. BMC Oral Health. (2020) 20(1):1–10. 10.1186/s12903-020-01323-z PMC7678153 33213442

[20] Leitão-Almeida B Camps-Font O Correia A Mir-Mari J Figueiredo R Valmaseda-Castellón E. Effect of bone loss on the fracture resistance of narrow dental implants after implantoplasty. An *in vitro* study. Med Oral Patol Oral Cir Bucal. (2021) 26(5):e611–8. 10.4317/medoral.24624 34162823 PMC8412446

[21] Shah SD Zheng F Seghi RR Lee DJ. Strength of titanium-zirconium alloy implants with a conical connection after implantoplasty. J Prosthet Dent. (2022) 132:593–9. 10.1016/j.prosdent.2022.08.015 36150928

[22] Sivolella S Brunello G Michelon F Concheri G Graiff L Meneghello R. Implantoplasty: carbide burs vs diamond sonic tips. An *in vitro* study. Clin Oral Implants Res. (2021) 32(3):324–36. 10.1111/clr.13702 33341106

[23] Tsampli A Rues S Kappel H Rammelsberg P Kappel S. *In vitro* pilot study comparing a novel implantoplasty sonic instrumentation protocol with a conventional protocol using burs. Clin Oral Implants Res. (2024) 35(3):340–9. 10.1111/clr.14231 38225734

